# ChIPnorm: A Statistical Method for Normalizing and Identifying Differential Regions in Histone Modification ChIP-seq Libraries

**DOI:** 10.1371/journal.pone.0039573

**Published:** 2012-08-03

**Authors:** Nishanth Ulhas Nair, Avinash Das Sahu, Philipp Bucher, Bernard M. E. Moret

**Affiliations:** 1 Laboratory for Computational Biology and Bioinformatics, School of Computer and Communication Sciences, École Polytechnique Fédérale de Lausanne (EPFL), Lausanne, Switzerland; 2 Department of Computer Science, University of Maryland, College Park, Maryland, United States of America; 3 School of Life Sciences, École Polytechnique Fédérale de Lausanne (EPFL), Lausanne, Switzerland; 4 Swiss Institute for Bioinformatics, Lausanne, Switzerland; National Institutes of Health, United States of America

## Abstract

The advent of high-throughput technologies such as ChIP-seq has made possible the study of histone modifications. A problem of particular interest is the identification of regions of the genome where different cell types from the same organism exhibit different patterns of histone enrichment. This problem turns out to be surprisingly difficult, even in simple pairwise comparisons, because of the significant level of noise in ChIP-seq data. In this paper we propose a two-stage statistical method, called ChIPnorm, to normalize ChIP-seq data, and to find differential regions in the genome, given two libraries of histone modifications of different cell types. We show that the ChIPnorm method removes most of the noise and bias in the data and outperforms other normalization methods. We correlate the histone marks with gene expression data and confirm that histone modifications H3K27me3 and H3K4me3 act as respectively a repressor and an activator of genes. Compared to what was previously reported in the literature, we find that a substantially higher fraction of bivalent marks in ES cells for H3K27me3 and H3K4me3 move into a K27-only state. We find that most of the promoter regions in protein-coding genes have differential histone-modification sites. The software for this work can be downloaded from http://lcbb.epfl.ch/software.html.

## Introduction

Histones are proteins that package the DNA into chromosomes [1]. They are subjected to various types of modifications like methylation, acetylation, phosphorylation, and ubiquitination, which alter their interaction with the DNA and nuclear proteins, thereby influencing transcription and genomic function. These modifications form an important category of epigenetic changes, changes that help us understand why various types of cells exhibit very different behaviors in spite of their shared genome. Thus the study of histone modifications, and more particularly of the differential enrichment of these modifications in different cell types, is a crucial tool in the understanding of genomic function. The current technology to capture histone modifications is chromatin immunoprecipitation (ChIP), which uses an antibody to isolate DNA fragments in contact with histones that carry a specific modification. ChIP-chip, ChIP-PET, and ChIP-SAGE are some of the ChIP-based technologies used for the study of histone modifications or transcription factor binding in genomic regions [2]–[4]. Thanks to advances in sequencing technologies, ChIP-seq has become the main approach for capturing histone modifications, due to its high throughput, high resolution, and low cost [5]–[7]. In the ChIP-seq process, the sequence of one end of the DNA fragment is read to provide a tag which is then mapped to an assembled genome to determine the location of the DNA fragment.

Genome-wide chromatin maps (using ChIP-seq technology) for three mouse cell types – embryonic stem (ES) cells, neural progenitor (NP) cells, and embryonic fibroblasts (EF) – have been published [8]. The authors of the paper compared the occurrence of histone-modification sites in promoter regions of the three cell types in a qualitative manner. Subsequently, the first quantitative comparison of two ChIP-seq libraries using computational techniques appeared [9]; there the authors addressed the problem of finding differential regions given two histone-modification libraries for two different cell types. Their method, ChIPDiff, is based on hidden Markov models. Recently, Taslim *et* *al.* 2009 [10] proposed a two-step nonlinear normalization method based on locally weighted regression (LOESS) [11] to compare ChIP-seq data across multiple samples; they modeled the difference using an exponential-normal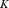 mixture model, then used this fitted model to identify genes associated with differential binding sites. Another recent method is RSEG [12]. From a mathematical viewpoint the problem of finding genomic regions with differential histone modifications between two tissues is not fundamentally different from that of peak finding in ChIP-seq data using an input control to correct for technical biases. Many such peak finders have been proposed [13].

A significant impediment to the analysis of ChIP-seq data is the high level of noise. Noise or systematic distortion can enter at various stages of the procedure: variations in the number of cells used in the experiment, variation in the amount of antibody that attaches to the DNA fragments, tandem repeats, uneven rates of success in sequencing different fragments, etc. The type of histone modification and the cell types can also affect the level of noise. For example, we show in this paper that histone modification H3K27me3 (K27) in ES cells has less background noise and a better signal-to-noise (S/N) ratio than the same modification in NP cells. In addition, the signal tends to be found mostly in gene-rich regions of the genome. Therefore computational methods may produce many false positives. In the case of modification K27 for ES and NP cells, for instance, false positives are likely in gene-poor regions for NP cells and in gene-rich regions for ES cells. Such bias problems are present in microarray data and many authors have addressed this issue [14]. Similar studies are needed in ChIP-seq data, as the data characteristics differ [15].

To address the problems of noise and bias in finding differential regions, we propose a two-stage statistical method, called ChIPnorm, to remove the noise and the bias from two ChIP-seq libraries and to normalize the data so as to enable a direct comparison between the two libraries to identify differential regions. Our normalization step is similar to quantile normalization [16]; however we have simplified the method so that it can be readily extended for normalization of more than two libraries. Our method is computationally efficient and can be applied to very large datasets. We use it to analyze ChIP-seq histone modification data from different types of mouse and human cells, confirming previous findings and making some new observations.

## Methods

**Figure 1:**
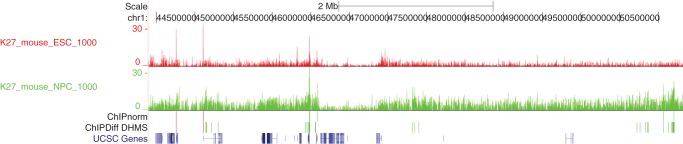
Histone modification profile as seen in the UCSC genome browser [**17**]. Tracks 1 (red) and 2 (green) show the H3K27me3 modifications for ES and NP cells, respectively. Track 3 shows the differentially enriched regions found by our ChIPnorm method. Track 4 shows the differentially enriched regions found by the ChIPDiff method [9]. In tracks 3 and 4, red indicates differential enrichment in ES cells and green indicates differential enrichment in NP cells. Track 5 shows the UCSC genes.

To motivate our work, we examine data for histone modification H3K27me3 (K27) in mouse ES and NP cells [8]. Figure 1 displays a window of the data mapped onto the mouse genome through the UCSC genome browser [17]. ES data has better S/N ratio as well as more peaks in gene-rich regions than in gene-poor regions. These characteristics introduce a bias that must be eliminated before comparing ES data to NP data, as can be seen in the results of the ChIPDiff method [9] in the same figure: most of the differentially NP enriched regions proposed by ChIPDiff fall within gene-poor regions and are almost certainly false positives.

In the following, we use a notation similar to that of Xu *et* *al.* 2008 [9]. In particular, we assume that the data has been processed by dividing the genome into bins and collecting, for each bin, a count of the mapped sequence tags that fall within the bin. The result is a “library”, which is simply a list of positive integers, each successive integer associated with the next bin. Let 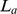 and 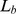 be two libraries containing the same histone modification for two different cell types – in our example, modification K27 for ES and NP cell types. Let 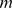 be the total number of bins in the library and set 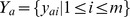 and 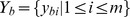 to be the observed counts of the ChIP-seq fragments for libraries 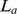 and 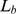 respectively, where 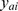, respectively 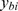, are the sum of the fragments lying in the 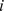
th bin. In ChIP-seq, a tag is retrieved by sequencing one end of the ChIP fragment, and the median length of this fragment is around 200 bp [5], [18]. As was done in Xu *et* *al.* 2008 [9], we approximate the center of each fragment by shifting the tag end position by 100 bp downstream or upstream, according to its orientation on the chromosome. We choose different bin sizes for different types of the histone modifications so as to maximize the discriminative information between the two libraries of the different cell types and minimize the discrimination of the two replicates of the same libraries. We use the spread of the data in the scatter plots as a measure for discriminative information. A lower bin size favors a better spread (away from the diagonal) between the data of the two cell types in the scatter plot.

An observed fragment count 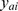 at the 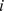
th bin can be related to the actual number of histone modifications 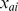 at the 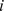
th bin using the following model:


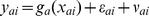



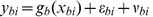
(1)

Function 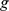 is the (unknown) deterministic function that describes the nonlinear transformation of the actual histone modifications, accounting for the various experimental conditions that may influence the observations in a systematic way. The additive 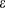 term accounts for the stochastic (background) noise introduced by the experimental setup, such as stray fragments from neighbouring modifications. Finally, the parameter 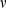 accounts for local genomic bias, mainly bias due to open chromatin regions and mapability, such noise is common in both the actual ChIP-seq library and the corresponding control dataset. Naturally, one could choose a stochastic, rather than a deterministic model for 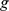; but since our goal is to detect regions with differential enrichment, and not to produce a detailed predictive model, the deterministic approach suffices.

### ChIPnorm scheme

We will address each source of error separately and proceed in two main stages. In the first stage, we address the removal of stochastic background noise and local genomic bias in each library. In the second stage, we address the problem of normalization.

### Stochastic noise 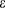


To solve the problem of stochastic background noise, both Bayesian modelling methods [19] and statistical confidence measure methods have been used. In terms of statistical confidence, the problem reduces to evaluating the probability that a particular bincount (bincount is the total number of DNA fragments captured inside a bin of some size) would occur by chance [20]. We estimate this probability by defining a “null hypothesis”, which is a random distribution of bincounts, and then comparing it with the distribution of bincounts of the ChIP-seq library.

**Figure 2:**
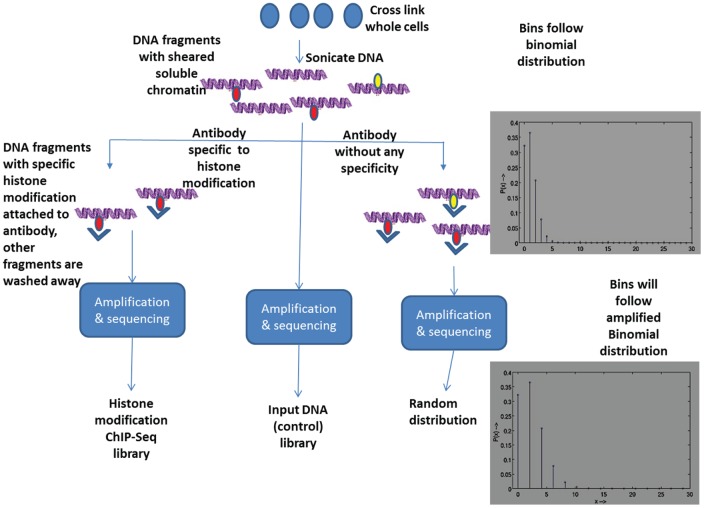
Overview of ChIP-seq process. We see how we can get the ChIP-seq library, input DNA control, and the random distribution (null hypothesis).

To understand the rationale for choosing the amplified binomial distribution (ABD) as the random distribution for the bincounts of ChIP-seq library, we must examine the ChIP-seq process as illustrated in Fig. 2. The short segments of DNA are treated with a specific antibody to capture a particular histone modification in the genome. The rest of the fragments are washed away. The captured fragments are sequenced by a high-throughput sequencing method, which typically uses PCR amplification of the captured fragments [21] before performing the final base-pair sequencing. The sequenced data is binned to obtain a ChIP-seq library.

To estimate the null distribution of a ChIP-seq library, we assume that the fragmented DNA from the sonicated whole cell is treated with an antibody that randomly captures fragments without any specificity. The bincounts of captured fragments then follow a binomial distribution. The captured fragments are amplified, sequenced, and binned to get the null distribution of the ChIP-seq library. This binned data follows a random distribution, in which each of the fragments following the binomial distribution is amplified; we refer to it as the amplified binomial distribution (ABD). We assume that each fragment is amplified by a constant amplification factor.

An ABD can be defined by two parameters: the total number of fragments 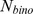 in its corresponding binomial distribution and the amplification factor 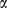. We made two assumptions to calculate these parameters for the desired null distribution: (a) the total number of the fragments in the original ChIP-seq data 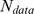 and in the corresponding null distribution 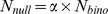 are the same, and (b) the total number of bins with zero bincount is the same in the ChIP-seq library (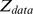) and the null distribution. Since the amplification does not change the number of bins with bin-count zero, we can write 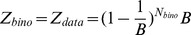, where 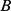 is total number of bins in the library. Since 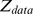 and 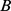 are observed variables, 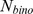 and 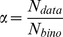 can be evaluated. Now the probability mass function of the random distribution prior to amplification is


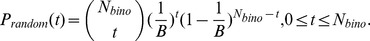


The ABD is thus estimated as 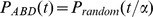.

We calculated the false discovery rate (FDR) for a ChIP-seq data using its corresponding estimated null distribution (ABD) [20]. We declared bins with FDR 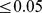 as significant bins in the ChIP-seq library. The value 0.05 is the standard value used in the field [20].

### Local genomic bias 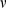


ChIP-seq data contains many local genomic biases corresponding to open chromatin regions, over-amplified satellite repeats, GC-rich regions, and unmappable perfect repeat regions. Some of these biases depend on experimental conditions and others may vary among the cell lines in a systematic manner. If such cell-specific biases are not taken care of while comparing ChIP-seq libraries it will give many false positive differential regions. Some of these biases can be reduced by using an input DNA control library. To find differential regions, we must consider only those regions that are significantly enriched with respect to the input DNA control. The input DNA control are the DNA fragments in the ChIP-seq experiment prior to the application of the histone specific antibody. To find the enriched bins, we need to normalize the input DNA control with respect to the data.

Yang *et* *al.* 2002 [22] recommended the use of locally weighted regression (LOESS) normalization. The basic assumption is that the percentage of the differential sites, considered as outliers by LOESS, is small so that these sites do not affect the normalization. However, this is not true in the case of a ChIP-seq library. In a ChIP-seq library, the percentage of bins that are differentially enriched relative to the input DNA control can exceed 50%. These differentially enriched bins will affect the LOESS normalization as shown in Figure S1(a) and lead to many false negatives. We introduce an iterative normalization to overcome this problem.

In the first stage of iterative normalization, we normalize the DNA control with respect to the data using quantile normalization. For illustration purposes, we first show the LOESS curve of the MA plot in Figure S1(a). We first classify bins enriched with respect to the DNA control (fold change 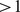) after quantile normalization as outliers. These outliers are then removed from further iterations. Figure S1(b) shows the LOESS curve after removing the outliers. We see that the LOESS curve is relatively less affected by outliers. In the next iteration, we use non-outliers bins for second quantile normalization to get a more accurate estimate of the normalization function. Once we get this normalization function, we normalize (quantile) all bins. The process of removing outliers and then performing normalization can be repeated to rescue more bins falsely declared as non-enriched. Each bin is declared as enriched if its fold-change value of ChIP-seq data and quantile normalized control data is 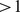. (The LOESS normalization is not used in the ChIPnorm method but is shown here only for illustrating the affects of normalization and removing outliers. Instead quantile normalization is used).

Bins which are declared both as significant in the ABD approach and as enriched in the iterative approach, are declared as ‘enriched-significant’. Bins which are declared as enriched-significant in either of the two libraries are passed on to the second stage, with their original bincount values.

### Quantile normalization

Since our first stage removed the majority of bins with low S/N ratios and genomic bias, and since we expect interesting regions to have good S/N ratios, we now make the simplifying assumption: 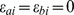 and 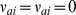.

With this assumption the next step is to normalize the data to the same scale so that bin values in the two libraries are comparable. Mathematically, given two observed data 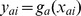 and 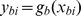, find a transformation 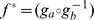 such that 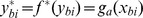.

**Figure 3:**
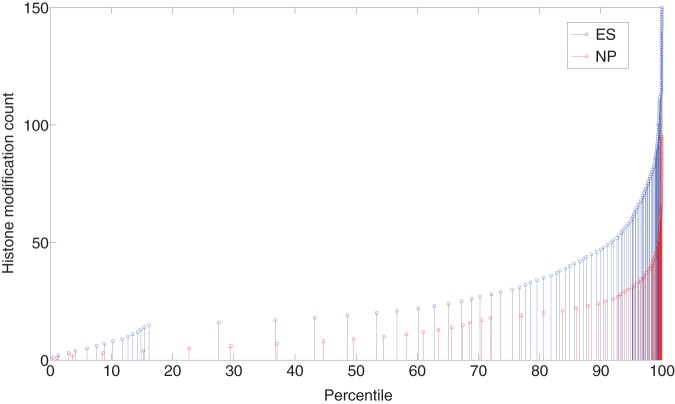
The new inverse cumulative distribution function on the modified libraries (after stage 1). On the 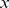 axis is the percentile, on the 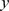 axis are the bin values.

We propose a quantile normalization method (similar to Bolstad *et al.* 2003 [16]) to solve this normalization problem. Quantile normalization assumes that the distribution of the data of the two libraries that are being compared are similar. This may seem problematic because histone modifications change significantly during differentiation. But it is reasonable to assume that their probability distribution of the bincount over the whole genome is similar across different cell types. (This might not be true if one of the two libraries have histone modifications knocked out.) We use the inverse cumulative distribution function (on the modified data after removing noisy bins) of the enrichment level, as shown in Fig. 3. The 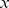 axis of this figure is the percentile while the 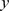 axis is the bin values. The figure shows the 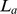 and 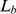 bin values plotted against their cumulative percentile. To get the desired transformation of 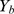, we must ensure that the post-transformation data 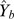 follows the same cdf as 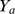. We fit a spline smoothing function on the bin values of library 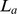, then, for all percentile values 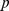, we perform a transformation 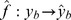 such that 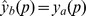. This is the desired transformation 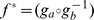. The proof is given in the supplementary material (Supporting Information S1).

This transformation reduces the problem of comparing two libraries with different probability distributions to the problem of comparing two libraries following the same probability distribution, so that a direct comparison of values can now be used. Since in the second stage we considered bins which were declared as enriched-significant in either of the two libraries 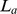 and 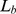 (a union operation), some bins which are not declared as enriched-significant would be present in the second stage too. If both libraries were completely independent events, we would expect 50% of the bins to be enriched-significant, because of the union operation. In effect, we define a bin in library 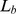 to be differentially enriched for the target modification if (i) observed bin value in libraries 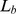 lies above the 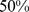 region in the inverse cumulative distribution function and (ii) for some chosen fold change threshold 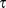 (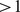), we have 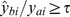. Similarly we can define a bin in library 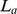 as differentially enriched if the bin value in library 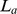 lies above the 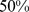 region in the inverse cumulative distribution function and for for some chosen threshold 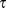 (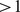), we have 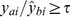. All bins are thus reported as differentially enriched or not. Adjacent bins of the same type of differential enrichment can be grouped together to form differential regions (DHE).

### The complete ChIPnorm method summarized

**Figure 4:**
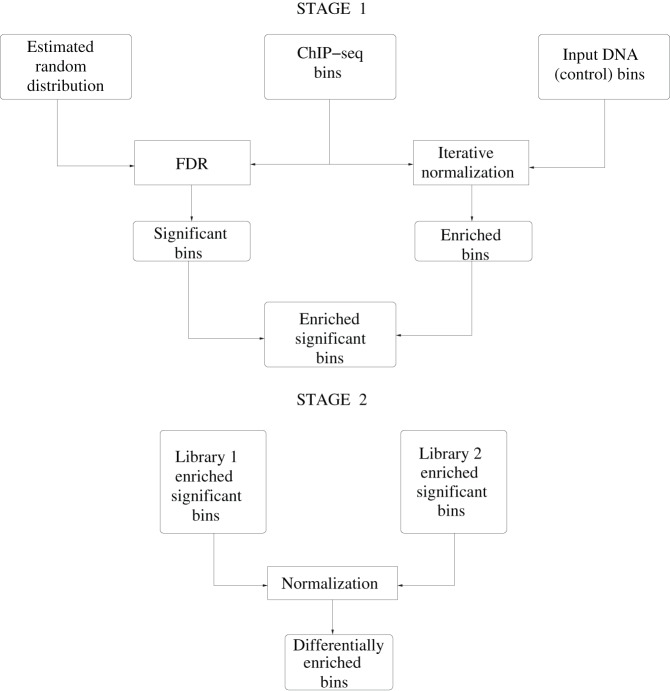
The schematic diagram of the ChIPnorm method. In the first stage one we find the enriched-significant bins by removing various kinds of errors in the data. In the second stage we normalize the two ChIP-seq libraries and find differentially enriched bins.

The complete ChIPnorm method is summarized in the Fig. 4. In the first stage, we identify bins having a significant bincount compared to the estimated random distribution of a ChIP-seq library as significant bins, by using a false discovery rate (FDR) analysis. We also identify bins of a ChIP-seq library as enriched bins, if their bincounts are higher than the corresponding bincounts of the normalized input DNA control. Those bins which are both significant w.r.t. null hypothesis and enriched w.r.t. normalized input control DNA are declared as enriched-significant bins. Bins which are declared as enriched-significant in either of these two libraries are passed to the second stage. In the second stage, we normalize the enriched-significant bincounts of the two ChIP-seq libraries and use a fold change to obtain differentially enriched bins.

The normalization can also be used to find bins that are enriched in both libraries, thereafter called constitutively highly enriched (CHE). Bins which are above 50% in 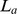 and 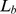 in the inverse cumulative distribution function and also below threshold 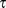 are declared as CHE. While differential histone modification enrichment (DHE) regions help us understand why different types of cells behave differently, CHE regions are conserved between the cell types and thus presumably essential to the survival of both types.

The normalization method also facilitates the comparison of more than two libraries. Our method is easily extended to handle multiple types of histone modifications in multiple cell types. Such analyses can give more insight into combinatorial patterns of histone modifications, sometimes referred to as “histone language” [23]. For example, Bernstein *et* *al.* 2006 [24] hypothesized that a bivalent domain with both H3K27me3 and H3K4me3 modification at the same site plays a crucial functional role in embryonic stem cells. Finally, the ChIPnorm method can be used with any ChIP-seq data – not just with histone modifications.

### Experimental Design

We carried out a series of experiments with the two libraries for H3K27me3 and H3K4me3 histone modifications (ES and NP cells), including experiments for bias and sensitivity. Since H3K4me3 has sharper peaks than H3K27me3, it needs a finer resolution, and smaller bin sizes are used. Using the replicate data analysis described earlier, we chose a bin size of 1000 bp for H3K27me3 (K27) and a bin size of 200 bp for H3K4me3 (K4). The bin size of 1000 bp for H3K27me3 has also been used previously in literature [9]. We compared ChIPnorm with six other normalization methods: (a) unit mean normalization; (b) quantile normalization; (c) MACS peak finder; (d) ChIPDiff method [9]; (e) rank normalization; and (f) two-stage unit mean normalization. We ran these methods on the H3K27me3 data for ES and NP mouse cells provided by Mikkelsen *et* *al.* 2007 [8] (with whole cell extract (WCE) control library) and on the H3K27me3 data (of Broad Institute) for ES and GM12878 (replicate 1) from the human ENCODE project [25], [26]. (GM12878 is a lymphoblastoid cell line produced from the blood of a female donor with northern and western European ancestry by EBV transformation [25].) Processing was done on individual chromosomes of the two libraries.

The five methods not yet described are as follows:

• *unit mean normalization*: is the standard Affymetrix scaling method for microarray data [16]. To perform consistent comparison with the ChIPnorm method, we normalized the two libraries to have unit mean using a method similar to the Affymetrix scaling method. To normalize the bin values 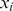 of a library we calculated its trimmed mean 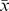 (the mean of the non-zero bins in the library) and then the normalized bin value is set to 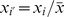. Finally a threshold (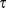) was used to classify bins as differential or not.
• *quantile normalization*: the two libraries are quantile normalized, and a fold change threshold (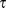) is used to classify bins as differential or not.
• *MACS peak finder method*: Although MACS is a peak-finding software [13], we use it indirectly to find differential regions as follows: peaks for one library are detected by giving the other library as control, and the bins with peaks are considered as differential regions.
• *rank normalization*: the bin values of each of the libraries are sorted separately; the sorted lists are divided into 10 equal partitions, which we define as rank. Finally we compare the values of corresponding ranks at each bin value in both libraries. If the difference between the values is greater than a threshold 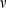 then the bin is classified as differential.
• *RSEG method:* RSEG is a recently published method [12] to not only find peaks in histone modifications but to also identify differential regions (rseg-diff) between two histone modification ChIP-seq libraries.
• *two-stage unit mean normalization*: we removed the noisy bins using the first stage of the ChIPnorm method before applying the unit mean normalization and fold change classification.

More details are given in the supplementary material (Supporting Information S1).

## Results

Our results are of two kinds. First, we present the characteristics of the ChIPnorm method and compare it to various other methods for normalization. Next, we use the ChIPnorm method to investigate the libraries of various cell types, both to confirm existing findings and to evince new correlations.

### Comparative Analysis

#### Bias with respect to gene density

**Figure 5:**
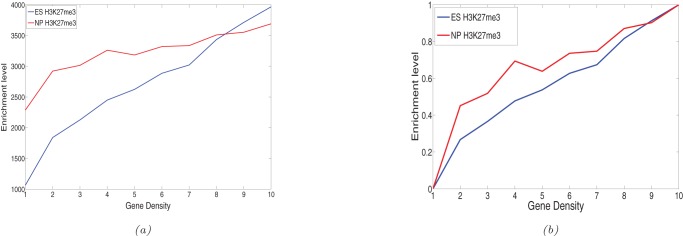
Enrichment level of bins with respect to gene density in a 1 Mbp region. The 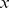 axis indicates lowest to highest gene density. (a) The 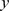 axis indicates the total number of H3K27me3 ChIP-seq fragments divided by the number of Mbp regions (counts per megabase) found in each gene density. (b) The plots are re-normalized so that the 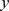 axis range is same for both ES and NP cell data. We see that the enrichment level of ChIP-seq data increases with respect to gene density for both ES and NP cells.

We now look how the number of histone modifications change with respect to gene density. Basically gene-rich regions are those regions of the genome where there are large number of genes. Even regions of the genome which are upstream and downstream of genes will fall into gene-rich/poor regions depending on the number of genes in that region. Usually the number of histone modifications is comparatively small in long stretches of the genome which have less or no genes (inactive regions). Gene poor regions have a high number of inactive regions. We noticed that most of the earlier methods for comparing two libraries suffer from bias with respect to gene density. In order to study this bias quantitatively, we divided the whole genome (data from Mikkelsen *et* *al.* 2007 [8]) into regions of size 1 Mbp each. The size of 1 Mbp was chosen so that there are sufficient number of genes within each region and also so that each region is not too big. Each of these regions is then classified into 10 classes according to the number of genes present in that region. Then we compared the number of bins declared enriched by previous methods and by the ChIPnorm method. Histone modifications like H3K27me3 and H3K4me3 mostly occur near the promoter regions of genes. Therefore there should be more differentially enriched regions in gene-rich regions than in gene-poor regions. First we give evidence that there are more histone modifications in gene-rich regions than gene-poor regions. Fig. 5(a) shows the total number of H3K27me3 ChIP-seq fragments divided by the number of Mbp regions (counts per megabase) found in each gene density. We see from the figure that there is an increasing trend of ChIP-seq fragments with gene density. This is true for both ES and NP cells. We also notice that the curve for ES cells is steeper than the curve for NP cells. This shows that NP cells have more background noise than ES cells and a lower signal-to-noise ratio. We think that the positive correlation of H3K27me3 levels and gene density is biologically meaningful: the more genes, the more gene regulatory regions that are potential targets of H3K27me3-mediated repression. From this perspective, the reversal of the trend in the top two gene-rich bins is due to the stronger “signal” (higher number of ChIP-Seq fragments originating from truly H3K27me3-enriched regions) in ES cells.

**Figure 6:**
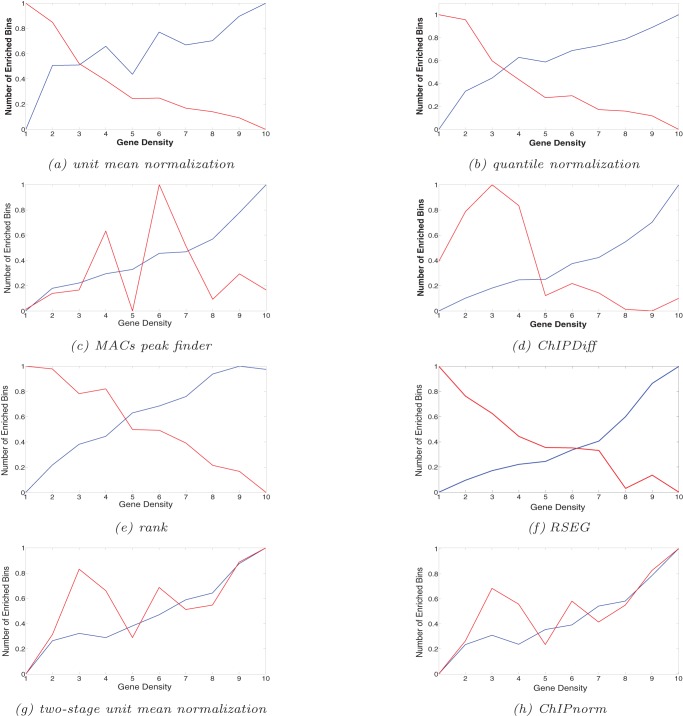
Enriched bins with respect to gene density in 1 Mbp region. The plots are normalized. 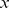 axis 1 to 10 indicates lowest to highest gene density, while 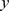 axis 0 to 1 indicates minimum to maximum average number of differentially enriched bins for both ES and NP cells. Blue line indicates ES differentially enriched bins and red line indicates NP differentially enriched bins. The number of enriched bins per 1 Mbp should increase with gene density.

Now we show the bias of various methods with respect to gene density. Figure 6 shows that other methods, namely unit-mean normalization (6(a)), quantile normalization (6(b)), ChIPDiff (6(d)), rank normalization (6(e)), and RSEG method (6(f)) all follow the trend for ES differentially enriched bins, but show an opposite trend for NP differentially enriched bins (For MACs peak-finder method (6(c)) the trend for NP with respect to gene density is not exactly opposite but more random). Because of the increased background noise and lower signal-to-noise ratio in NP data compared to ES data, these methods incorrectly show an increased number of H3K27me3 NP differentially enriched regions in gene-poor regions. This is due to the ineffective normalization techniques. This ineffectiveness is removed by the first stage of the ChIPnorm approach, as it removes the noisy or insignificant bins for each ChIP-seq library separately. The unit-mean and quantile normalization (Fig. 6 (a) and (b)) methods shows decreasing trends in gene density for NP cells, but after applying the first stage of ChIPnorm, this trend is reversed (Fig. 6 (g) and (h) for two-stage unit-mean normalization and ChIPnorm). This shows the importance of the first stage of the ChIPnorm method, which is common to both these approaches.

#### Sensitivity analysis

From the 13,438 genes whose microarray gene expression data is available in Mikkelsen *et* *al.* 2007 [8], we selected genes for which expression levels are at least four-fold upregulated in ES cells compared to NP cells, and vice-versa. Out of 13,438 genes, 925 genes were at least four-fold differentially over-expressed in NP cells, and 1104 genes were at least four-fold differentially over-expressed in ES cells. We carried out sensitivity analysis on these genes on the various methods. Since K27 is thought to be a gene repressor [5], [8], we expect that there are more ES enriched differential regions in the promoter region (which we define here as 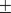
1 kbp of the transcription start site (TSS)) of those genes which are over-expressed in NP cells, and more NP enriched differential regions in the promoter region of genes which are over-expressed in ES cells. Note however that we do not expect a high correlation in such a test, as H3K27me3 modification of histone H3 is only one of several mechanisms of gene repression. We are also aware of recent work that questions the exclusively repressive role of H3K27me3 in gene regulation. Young *et* *al.* 2011 [27] identified a new subclass of H3K27me3-marked genes, which are highly expressed. However, as these genes were reported to have unchanged expression levels between ES and NP cells in the same paper, they are unlikely to interfere with our evaluation protocol.

The results of our sensitivity analysis are summarized in Table 1 (data from Mikkelsen *et* *al.* 2007 [8]). The TSS positions were taken from the “knownGene” track of the UCSC genome browser. For each gene all the promoters were considered. Experiment “sensitivity (ES K27-enriched)” shows the percentage of ES differentially enriched regions around the TSS 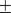
1 kbp of the 925 genes which are at least four-fold over-expressed in NP cells. Experiment “error (NP K27-enriched)” was done on the same 925 genes as experiment “sensitivity (ES K27-enriched)”, but these regions were erroneously declared as NP-enriched instead of being declared as ES-enriched. Likewise, “sensitivity (NP K27-enriched)” and “error (ES K27-enriched)” was determined for the 1104 genes over-expressed in ES cells. To compare the output of the various methods we fixed the parameters so that each of the methods give similar sensitivity for ES K27-enriched regions (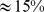). The MACS peak-finder method could not be made to get a sensitivity close to 15% by changing the p-value threshold. We see that all methods give a low “error (NP K27-enriched)” rate which shows that all methods have a good one-sided accuracy. However, we see that for the same thresholds, unit-mean, quantile normalization, MACS peak-finder and rank normalization methods show very small sensitivity for ES differentially over-expressed genes (“sensitivity (NP K27-enriched)”) and a corresponding high error rate (“error (ES K27-enriched)”). In fact, we find that the error rates are higher in the quantile normalization method, the MACS peak-finder method, the ChIPDiff method, the RSEG method, than the sensitivity for ES differentially over-expressed genes. This clearly shows a bias towards ES enrichment in promoter regions. However, for the two-stage unit mean and the ChIPnorm method, the problem of bias disappears, as these methods give a higher sensitivity and lower error rate on both the ES and NP differentially over-expressed genes. The reason the ChIPnorm method, improves over the other methods is because the first stage of the ChIPnorm approach, removes the noisy regions, while the second stage which uses quantile normalization, transforms one distribution of one library to that of the other library, thereby reducing the differences in the amplification factors and the SNR of the two libraries. The two-stage unit mean method also works well showing the importance of the first stage that we used, which is common in both these methods.

**Table 1:** Sensitivity analysis percentages using various methods (data from Mikkelsen et al. 2007 [8]).

	unit-mean	quantile	MACS	ChIPDiff	rank	RSEG	two-stage unit-mean	ChIPnorm
thresholds	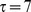	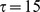	p-val 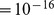	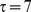	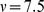	cdf 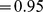	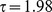	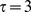
NP differential (four-fold) expressed: 925 genes								
sensitivity (ES K27-enriched)	14.49	15.24	31.24	14.64	17.62	18.16	15.14	15.14
error (NP K27-enriched)	0	0	0	0.27	6.05	0.11	0	0
ES differential (four-fold) expressed: 1104 genes								
sensitivity (NP K27-enriched)	0	1.27	0.27	0	12.59	1.9	6.88	7.16
error (ES K27-enriched)	1.27	1.99	5.07	0.91	5.43	3.08	1.18	1.99

Experiments: unit-mean; quantile; MACS peak finder; ChIPDiff; Rank normalization; two-stage unit-mean; ChIPnorm. The parameters of all the methods (except MACS) were adjusted so that all of them give almost the same percentage (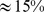) of experiment “sensitivity (ES K27-enriched)”.

**Figure 7:**
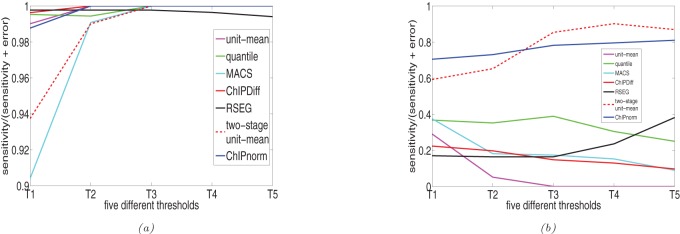
Plot of 
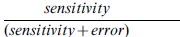
 over five different threshold values. (a) NP is differentially over-expressed compared to ES, (b) ES is differentially over-expressed compared to NP.

Since the above sensitivity analysis was done by fixing the sensitivity value to 15%, we tested the various methods over a wide range of thresholds. Figure 7 gives the plot of 
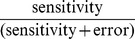
 over five different threshold values (T1, T2, T3, T4, T5) in increasing order. The actual values of these five thresholds for each method is given in the supplementary material (Supporting Information S1). The higher the value of this ratio, the better the method works, as it shows the error is less. Figure 7(a) gives the plots for the case when NP is differentially over-expressed compared to ES while 7(b) shows the case when ES is differentially over-expressed compared to NP. We see from the plots that although most methods work well when NP is differentially over-expressed, only the two-stage unit mean normalization and the ChIPnorm method works well when ES is differentially over-expressed compared to NP. In fact the other methods show a ratio less than 0.5 (Figure 7(b)), indicating that the error is greater than sensitivity. This clearly indicates that the first stage of the proposed ChIPnorm approach helps remove the problem of bias which happens because of the different signal-to-noise ratios in the two libraries.

**Figure 8:**
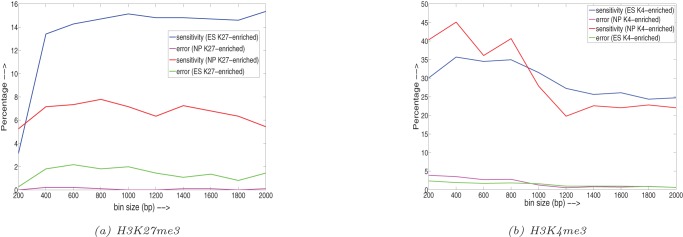
Robustness studies: sensitivity and error analysis for ChIPnorm by fixing the fold-change threshold 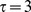 and varying the bin size from 200 bp to 2000 bp in steps of 200 bp. Data from Mikkelsen et al. 2007 [8].

In this paper, we used a bin size of 1000 bp for H3K27me3 data and 200 bp for H3K4me3 data. However we have done robustness studies by varying bin sizes. The sensitivity and error experiments shown in table 0 for the data in Mikkelsen *et* *al.* 2007 [8] are repeated here for the ChIPnorm approach by varying the bin sizes for H3K27me3 and H3K4me3 data. The fold change threshold (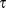) is fixed at 3 (as done in Table 1) for the ChIPnorm method and the bin sizes vary from 200 bp to 2000 bp in steps of 200 bp. We see from Fig. 8 that the sensitivity and error vary little for bin sizes varying from 400 bp to 2000 bp for H3K27me3 and 200 bp to 800 bp for H3K4me3. (For H3K4me3 data, while the sensitivity drops after 800 bp bin size, so does the error.) This shows that the ChIPnorm method is robust over a wide range of bin sizes and that there is a large range of bin sizes where ChIPnorm works well for both H3K27me3 and H3K4me3 data.

We repeated the sensitivity tests for the human data from the ENCODE Broad database (hg18). We tested the experiments on ES and GM12878 cell lines for H3K27me3 histone modifications [25]. The corresponding gene expression data (RPKM) is from the ENCODE Caltech RNA-seq database [25]. The parameters of all the methods (except rank normalization) were adjusted so that all of them give almost the same percentage (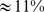) of experiment “sensitivity (HES K27-enriched)”. For the rank normalization method we could not further change the threshold to get a sensitivity close to 11%. The results are summarized in Table 2. We can clearly see that ChIPnorm and the two-stage unit-mean approach outperform all other methods. However the two-stage unit mean approach results vary a lot based on the thresholds. When the threshold 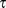 is changed from 1.4 to 1.89, so as to fix “sensitivity (GM12878 K27-enriched)” to approximately the same as that of ChIPnorm approach (i.e. approximately 12%), the results are: “sensitivity (GM12878 K27-enriched)” is 12.07% while the corresponding “error (HES K27-enriched)” is 0.86%; “sensitivity (HES K27-enriched)” reduces to 4.67% (from the earlier 10.69% for 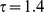) while the corresponding “error (GM12878 K27-enriched)” value is 0.21%. Therefore the sensitivity of the two-stage unit-mean approach (“sensitivity (HES K27-enriched)”) reduced with change in threshold. In fact for this data set, ChIPnorm results varies very little with change in threshold. For example, for ChIPnorm when we change the threshold 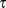 from 3 to 15, the “sensitivity (HES K27-enriched)” values change from 12.71 to 10.07. Similarly the rest of the results do not vary much. This leads us to believe that the second stage quantile normalization approach in ChIPnorm, gives stabler results as it equates the distributions of the two libraries.

**Table 2:** Sensitivity analysis for human ES and GM12878 cells (replicate 1 data from ENCODE Broad database) percentages using various methods.

	unit-mean	quantile	MACS	ChIPDiff	rank	RSEG	two-stage unit-mean	ChIPnorm
thresholds	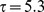	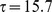	p-val 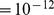	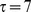	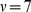	cdf 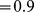	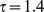	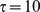
GM12878 differential (four-fold) expressed: 1927 genes								
sensitivity (HES K27-enriched)	11.26	11.26	11.26	10.85	17.85	8.27	10.69	11.05
error (GM12878 K27-enriched)	0.10	0.21	0	0.16	5.35	0	0.31	0.16
HES differential (four-fold) expressed: 2908 genes								
sensitivity (GM12878 K27-enriched)	1.55	10.97	1.79	4.09	33.29	0.06	14.65	12.00
error (HES K27-enriched)	1.38	0.93	3.54	1.20	2.27	11.81	1.20	0.55

Experiments: unit-mean; quantile; MACS peak finder; ChIPDiff; Rank normalization; two-stage unit-mean; ChIPnorm. The parameters of all the methods (except rank normalization) were adjusted so that all of them give almost the same percentage (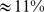) of experiment “sensitivity (HES K27-enriched)”.

### ROC curves

We next plot receiver operating characteristics (ROC) for H3K27me3 histone modification data (Mikkelsen *et* *al.* 2007 [8]) to compare the various techniques. Since what is a ‘true’ differential region is unknown, we used indirect ways of calculating true positives (TP), true negatives (TN), false positives (FP), false negatives (FN), by comparing the results with gene expression data. For the sake of plotting the ROC curves, we convert the 3-sided testing problem (ES differentially enriched, NP differentially enriched, or non-differential) into a two-sided problem. We plot two different ROC curves and define the various parameters keeping in mind that K27 is a repressor.

## For the first ROC

Class 1 is defined as four-fold NP differentially over-expressed genes compared to ES; Class 0 is rest of the genes.

TP – genes were ES (ChIP-seq) is declared differentially enriched (i.e. above threshold) in Class 1 (since H3K27me3 is a repressor).

FN – rest of the genes which fall in Class 1.

FP – genes were ES (ChIP-seq) is declared differentially enriched (i.e. above threshold) in Class 0.

TN – rest of the genes which fall in Class 0.

True positive rate (TPR)  =  sensitivity  =  TP/(TP + FN).

False positive rate (FPR)  =  (1 – specificity)  =  FP/(FP+ TN).

## For the second ROC

Now Class 1 is defined as four-fold ES differentially over-expressed genes compared to NP; Class 0 is rest of the genes. And the rest of the parameters are defined for NP differentially enriched (opposite of the previous case).

**Figure 9:**
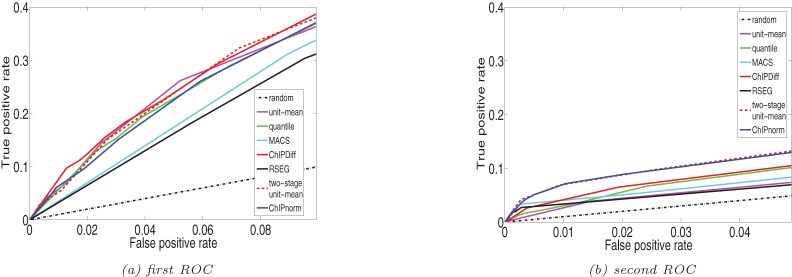
Two ROC curves are shown for various methods. (a) first ROC: Class 1 – four-fold NP differentially over-expressed genes compared to ES; Class 0: rest of the genes. (b) second ROC: Class 1 – four-fold ES differentially over-expressed genes compared to NP; Class 0: rest of the genes.

The two ROC curves are shown in Figure 9. It is important to note that this is only an approximate way of calculating ROCs as gene expression depends on more than just H3K27me3 histone markers but also on many other factors (like other histone modifications, transcription factors, etc.). Therefore a 100% TPR is not necessarily a good result. We varied the thresholds for various methods. In some methods, the values of TPR and FPR do not go beyond a certain value, irrespective of the thresholds. Therefore we show the plots of the regions where maximum value of the FPR exists for all methods. From the figure, it is seen that most methods work well for the first ROC, while for the second ROC curve, ChIPnorm and two-stage unit mean normalization outperforms all other methods, clearly showing the removal of the one sided bias.

### False-positive rate

Since the ENCODE Broad database for human histone modifications data [25] has a two replicates for each cell type, we did a false-positive rate study on this data. Basically we ran the methods on H3K27me3 libraries for ES cells for replicates 1 and 2. Since the two libraries used are replicates, we do not expect any differentially enriched regions between the libraries. So any bin declared as differential is considered a false positive. Since the false-positive rate is dependent on the thresholds used, we used the same thresholds as used in Table 2 for the various methods. The results are shown in Table 3. We see that the false-positive rates are below 1% in all the methods (except rank normalization) and for ChIPnorm it is very close to 0%.

**Table 3:** False-positive rate (FPR) analysis for human ES cells (H3K27me3 data from ENCODE Broad database) for the two replicates.

	unit-mean	quantile	MACS	ChIPDiff	rank	RSEG	two-stageunit-mean	ChIPnorm
thresholds	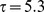	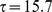	p-val 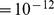	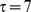	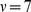	cdf 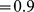	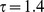	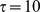
FPR	0.3924	0.0004902	0.0053	0	8.2753	0.4845	0.8652	0.0088

We see the percentage of false positive using various methods. Experiments: unit-mean; quantile; MACS peak finder; ChIPDiff; Rank normalization; two-stage unit-mean; ChIPnorm. The thresholds used are same as those in Table 2.

## Correlation with gene expression

We analyze the effect of presence of K27 and K4 sites (mouse data from Mikkelsen *et* *al.* 2007 [8]) on gene expression levels. K4 is associated with activation of genes, while K27 is associated with repression [8]. 13,000 UCSC known genes are used for this purpose. We divide these genes into five groups (A–E) according to the increasing log ratio of their expression levels in ES and NP cells. We take into account the distribution of the number of genes with respect to the log ratio of their expression levels to make the division. This grouping ensures enough representation in each group. Genes in each group are further classified for each K27 or K4 according to the presence of modifications in the promoter (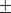
1 kbp of TSS) region. These categories are: type 1 genes have neither DHE nor CHE bins in their promoter regions; type 2 genes have at least one DHE bin enriched for ES cells, but not even one CHE or DHE bin with NP enrichment; type 3 genes have at least one CHE bin or at least two bins with opposite enrichments; type 4 genes have at least one DHE bin enriched for NP cells, but no CHE or DHE bin enriched for ES cells.

**Figure 10:**
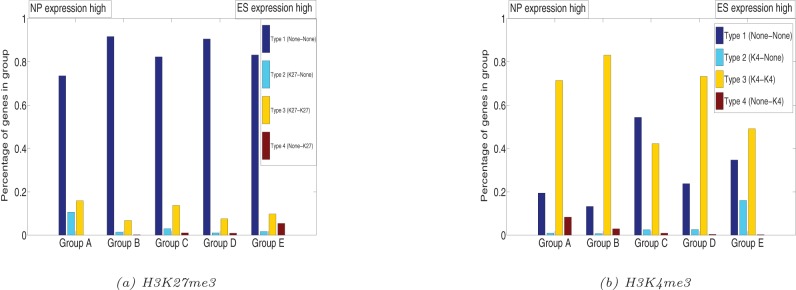
Gene profile according to expression and histone modifications. Genes are grouped in (A–E) according to increasing ratio of expression level in ES cells and NP cells. Within each groups, genes are classified into 4 types. Type 2 genes have differential histone enrichment in ES cells in their promoter regions and type 4 genes have differential enrichment in NP cells. (a) Percentage of type 2 genes is decreasing, while percentage of type 4 genes is increasing along the group (A–E). (b) Percentage of type 2 genes is increasing, while percentage of type 4 genes is decreasing along the group (A–E).

Figure 10(a) shows the percentages of genes in each group for H3K27me3. These percentages decrease from group A to group E, indicating that the number of genes differentially enriched for modification K27 in ES cells decreases at higher levels of differential gene expression. On the other hand, type 4 genes, which are differentially enriched for NP cells, increase from group A to group E. Thus we see clear evidence of negative correlation of K27 with gene expression, confirming the repressive regulation by K27. Similar conclusions can be drawn for K4 from Fig. 10(b), indicating the positive correlation of K4 with expression levels and thereby confirming its association with the activation of genes.

## Bivalent region analysis

H3K27me3 and H3K4me3 are sometimes present simultaneously at the same promoter [8], [24]. Such bivalent regions may repress the developmental genes in ES cells, while keeping them poised for activation at later stages of development in partially differentiated cells. Thus bivalent regions could play an important role in the maintenance of pluripotency for ES cells. We would therefore expect bivalent regions to be enriched in ES cells as compared to the better differentiated NP cells. We would also expect that bivalent regions in ES cells would preferentially lose the K27 rather than the K4 mark in NP cells.

**Figure 11:**
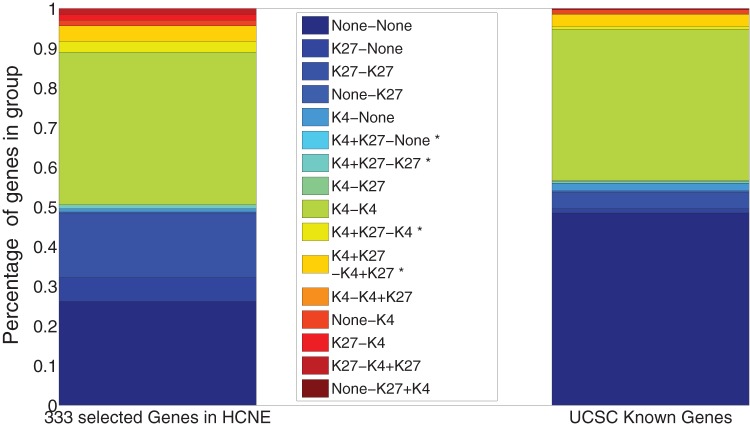
Bivalent regions in genes; (a) in 333 selected genes; (b) in UCSC known genes. Genes are attributed to classes according to the presence of modifications in ES and NP cells. The “A–B” notation in the labels indicates the presence of modification of type “A” in ES cells and modification of type “B” in NP cells. (*) marked labels have bivalent domains in ES cells.

We applied the ChIPnorm method to investigate these conjectures about bivalent regions in ES cells. First we selected 333 genes from chromosomal regions that are rich in highly conserved noncoding elements (HCNEs) which were previously analyzed by Bernstein *et* *al.* 2006 [24]. We classified these genes into 16 classes according to the presence or absence of K27 or K4 modifications in one or both of ES and NP cells (data from Mikkelsen *et* *al.* 2007 [8]). Figure 11(a) shows the representation of each class of genes among these selected HCNE genes.

The “A–B” in a label indicates the presence of modification “A” in ES cells and of modification “B” in NP cells; as before, detected but depleted modifications are treated as if they were absent; labels marked with an asterisk denote bivalent regions. We found that about 20% of these 333 genes had bivalent regions within 1 kb from the promoter in ES cells – a result consistent with Bernstein *et* *al.* 2006 [24]. We then examined the whole genome for the presence of bivalent regions. Figure 11(b) shows the representation of such classes among UCSC known genes. Though the percentage of genes with bivalent regions drops to about 10%, it remains surprisingly high, suggesting that bivalent histone marks in ES cells are not confined to key developmental regulators. We also note that bivalent marks specific to NP cells are extremely rare, while bivalent mark present in both cell types occur at an intermediate frequency. Contrary to our expectation, bivalent marks of ES cells do not preferentially lose their K27 mark. A substantial fraction loses the K4 mark instead, which may reflect a transition into a permanently repressed state.

**Figure 12:**
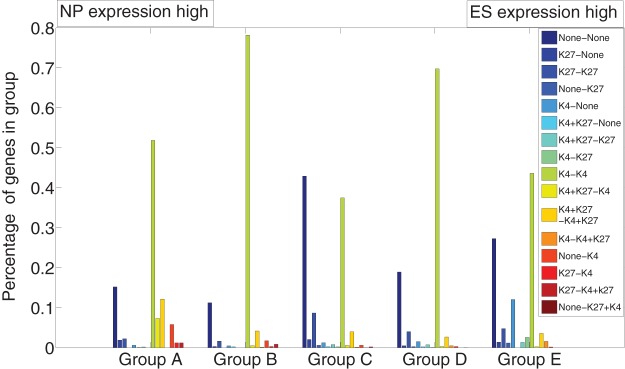
Bivalent gene profile vs. expression data. Genes are grouped in (A–E) according to increasing ratio of expression level in ES cells and NP cells. Each bar shows the percentage of genes with the corresponding “A–B” modifications (as listed in the box), “A” for modifications in ES cells and “B” for modifications in NP cells. It is seen that there is a strong over-representation of the K4+K27-K4 transition (yellow) in the genes class, which is strongly up-regulated in NP cells.

Finally we studied the connection between bivalent regions and gene expression levels. We divided the UCSC known genes in the same A–E groups according to the log ratio of expression levels in ES and NP cells. Within each class we further classified genes into 16 classes according to the presence of one or both histone modifications in ES and NP cells. Figure 12 shows a strong over-representation of the K4+K27-K4 transition in the class of genes that are strongly up-regulated in NP cells, indicating that the fate of a bivalent mark indeed influences the expression of the corresponding gene in a progenitor cell. Overall, our findings support the hypothesis of Bernstein *et* *al.* 2006 [24] that bivalent K4+K27 marks are frequent in ES cells and associated with a temporary repression of genes that need be activated later in development. Our results extend Berstein's hypothesis in that we show that bivalent marks are not confined to HCNE-associated key-regulatory genes, and that a sizable fraction of them transits into a K27-only state possibly reflecting permanent repression. Moreover, using our normalization method, we show that transition of a bivalent state into a K27-only state is a rather frequent event rather than an exception as reported in previous papers (e.g. Cui *et* *al.* 2009 [28]).

## Differential enriched regions along protein-coding genes

We analyzed the human ENCODE ES and GM12878 H3K27me3 ChIP-seq data [25] using Segtools [29]. When we looked at protein-coding genes, we found some evidence that most of the promoter regions have differentially enriched histone-modification sites, and very few non-differential sites. Please see supplementary material (Supporting Information S1) for details.

## Conclusion

We have presented an approach for the analysis of ChIP-seq data, with particular emphasis on the discovery of differentially enriched histone-modification sites. The problem of the bias inherent in the comparison of two sets of data with different noise backgrounds is biologically more relevant because such bias shows a false correlation between computationally identified differential regions with gene density. The ChIPnorm removes most of this bias and provides a normalization that enables direct comparison of values. We have conducted experiments that demonstrate that this new approach improves significantly on the state of the art. Finally, we have used our approach to highlight some aspects of K27 modifications in mouse embryonic stem cells and neural progenitor cells, including a so far unnoticed transition of bivalent mark of K4 and K27 in embryonic stem cell to a K27-only state in differentiated cells, possibly reflecting permanent repression of developmental genes. For the human ENCODE H3K27me3 data for ES and GM12878 cells, when we look at protein-coding genes, we provide evidence that most of the promoter regions have differentially enriched histone-modification sites. Recently, the ChIPnorm approach has also been used to study histone methylation changes associated with leaf senescence in Arabidopsis [30].

Our approach is not restricted to the identification of differentially enriched sites nor is it limited to pairwise comparisons. A natural next step, therefore, is to apply it to more complex data (multiple cell types with multiple histone modifications, for instance), to verify its efficacy, and to use it to shed light on the complex interactions described in the “histone language”.

## Supporting Information

Figure S1 Iterative normalization of input DNA. (a) before first iteration. (b) after first iteration, post removal of outliers.
(PDF)

Supporting Information S1 Supplementary material containing Supplementary figures, Supplementary table, Supplementary methods, Supplementary results, and Supplementary references [8], [9], [12], [13], [16], [25], .
(PDF)
