# Supplementary material for: ChIPnorm: A Statistical Method for Normalizing and Identifying Differential Regions in Histone Modification ChIP-seq Libraries
Source: PLoS One. 2012 Aug 3;7(8):e39573. doi: 10.1371/journal.pone.0039573 (PMC3411705; doi:10.1371/journal.pone.0039573)
Supplement: Figure S1 — Iterative normalization of input DNA. (a) before first iteration. (b) after first iteration, post removal of outliers. (PDF) [file pone.0039573.s001.pdf]

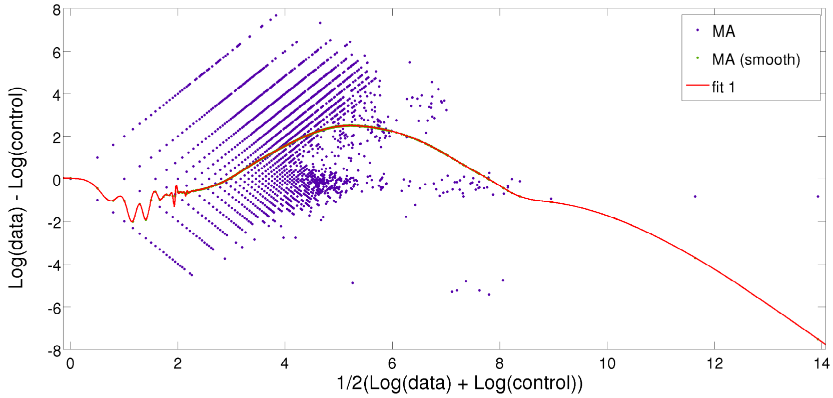

(a)

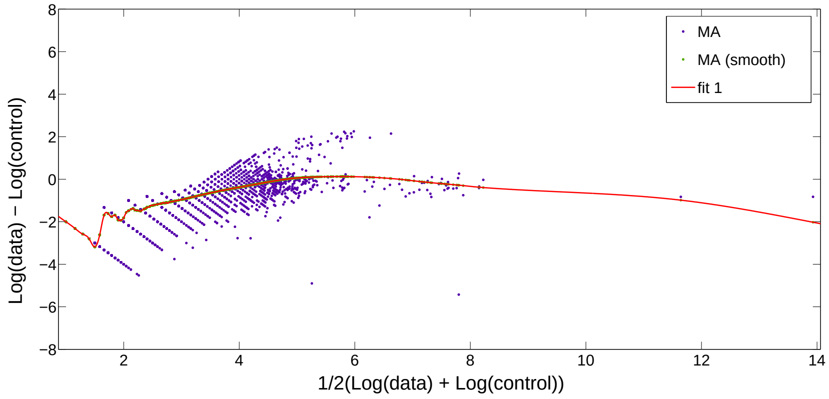

(b)

Figure S1. *Iterative Normalization of input DNA. (a) before first iteration. (b) after first iteration, post removal of outliers.*
