# Supplementary material for: ChIPnorm: A Statistical Method for Normalizing and Identifying Differential Regions in Histone Modification ChIP-seq Libraries
Source: PLoS One. 2012 Aug 3;7(8):e39573. doi: 10.1371/journal.pone.0039573 (PMC3411705; doi:10.1371/journal.pone.0039573)
Supplement: Supporting Information S1 — Supplementary material containing Supplementary figures, Supplementary table, Supplementary methods, Supplementary results, and Supplementary references [8], [9], [12], [13], [16], [25], . (PDF) [file pone.0039573.s002.pdf]

Supplementary material for paper "ChIPnorm: a statistical method for normalizing and identifying differential regions in histone modification ChIP-seq libraries"

Nishanth Ulhas Nair[1,2], Avinash Das Sahu[1,3], Philipp Bucher[4,5,*], and Bernard M.E. Moret[2,5,*]
1 who contributed equally to this work.
2 Laboratory for Computational Biology and Bioinformatics, School of Computer and Communication Sciences, École Polytechnique Fédérale de Lausanne (EPFL), Lausanne, Switzerland.
3 Department of Computer Science, University of Maryland, College Park, Maryland, USA.
4 School of Life Sciences, École Polytechnique Fédérale de Lausanne (EPFL), Lausanne, Switzerland.
5 Swiss Institute for Bioinformatics, Switzerland.
∗ E-mail: philipp.bucher@isb-sib.ch, bernard.moret@epfl.ch

METHODS

Quantile normalization

Since our first stage removed the majority of bins with low S/N ratios and genomic bias, and since we expect interesting regions to have good S/N ratios, we make the simplifying assumption:

$$\begin{aligned} \varepsilon_{ai} &= \varepsilon_{bi} = 0 \\ \nu_{ai} &= \nu_{ai} = 0; \end{aligned} \tag{1}$$

With this assumption the next step is to normalize the data to the same scale so that bin values in the two libraries are comparable. We propose a quantile normalization method (similar to Bolstad *et al.* 2003 [1]) to solve this normalization problem. We formulate the problem mathematically as follows. Given two observed data $y_{ai} = g_a(x_{ai})$ and $y_{bi} = g_b(x_{bi})$, find a transformation $f^* = (g_a \circ g_b^{-1})$ such that $y^*_{bi} = f^*(y_{bi}) = g_a(x_{bi})$. We make the following assumptions:

- The actual histone modifications $X_a = \{x_{ai} \mid i \in \{1, m\}\}$ and $X_b = \{x_{bi} \mid i \in \{1, m\}\}$ follow the same distribution, i.e., we have $F_{X_a}(x) = F_{X_b}(x)$.
- Cumulative distribution functions (cdf) F_{X_a} and F_{X_b} are monotonically increasing.
- $g_a(\ )$ and $g_b(\ )$ are monotonically increasing.

The last two conditions imply that F_{Y_a} and F_{Y_b} are also monotonically increasing.

Theorem 1
Any function $\hat{f} : y_{bi} \to \hat{y}_{bi}$ satisfies $F_{\hat{Y}_b}(y) = F_{Y_a}(y)$ if and only if we have $\hat{f} = f^*$.

Lemma 1 $\quad F_{Y^*_b}(y) = F_{Y_a}(y)$

Proof (of lemma):
$F_{Y_a}(y) = P(Y_a \le y) = P(g_a(X_a) \le y) = P(X_a \le g_a^{-1}(y)) = F_{X_a}(g_a^{-1}(y))$ and
$F_{Y^*_b}(y) = P(Y^*_b \le y) = P(g_a(X_b) \le y) = P(X_b \le g_a^{-1}(y)) = F_{X_b}(g_a^{-1}(y)) = F_{X_a}(g_a^{-1}(y))$

Proof (of theorem):
only if part: if we have $\hat{f} = f^*$, then from Lemma 1, we also have $F_{\hat{Y}_b}(y) = F_{Y^*_b}(y) = F_{Y_a}(y)$.

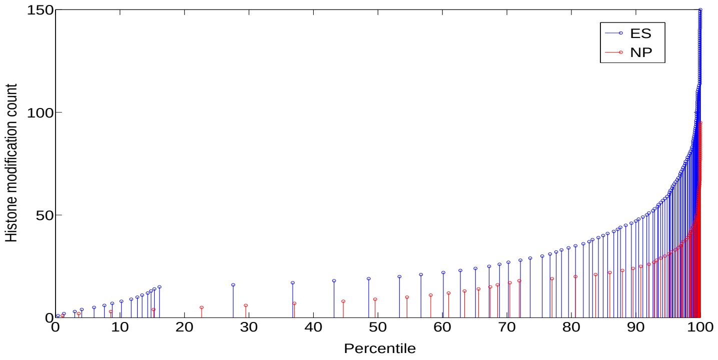

Figure F1. *The new inverse cumulative distribution function on the modified libraries (after stage 1). On the x axis is the percentile, on the y axis are the bin values.*

if part: if we have $F_{\hat{Y}_b}(y) = F_{Y_a}(y)$, then from Lemma 1, we also have $F_{Y_b^*}(y) = F_{\hat{Y}_b}(y)$. Additionally, as cdfs are assumed to be monotonically increasing, they are one-to-one functions. Hence we can write $\forall i$, $\hat{y}_{bi} = y^*_{bi}$, which in turn implies $\hat{f} = f^*$.

Theorem 1 states that if (i) we have $F_{X_a}(x) = F_{X_b}(x)$, (ii) the cumulative density functions of X_a and X_b are identical and monotonically increasing, and (iii) $g_a(\ )$ and $g_b(\ )$ are deterministic monotonic increasing functions, then any transformation that meets the conditions of the theorem is our desired transformation $f^* = (g_a \circ g_b^{-1})$.

To find such a transformation, we use the inverse cumulative distribution function (on the modified data after removing noisy bins) of the enrichment level, as shown in Fig. F1. The x axis of this figure is the percentile while the y axis is the bin values. The figure shows the L_a and L_b bin values plotted against their cumulative percentile. To get the desired transformation of Y_b, we must ensure that the post-transformation data $\hat{Y}_b$ follows the same cdf as Y_a. We fit a spline smoothing function on the bin values of library L_a, then, for all percentile values p, we perform a transformation $\hat{f}\colon y_b \rightarrow \hat{y}_b$ such that $\hat{y}_b(p) = y_a(p)$. The transformation $\hat{f}$ ensures that the conditions of Theorem 1 are met.

Experimental Design

We carried out a series of experiments with the two libraries for H3K27me3 histone modifications (ES and NP cells), including experiments for bias and sensitivity. The libraries were built with a bin size of 1000 base pairs. We compared ChIPnorm with six other normalization methods: (a) unit mean normalization; (b) quantile normalization; (c) MACS peak finder; (d) ChIPDiff method [2]; (e) rank normalization; and (f) two-stage unit mean normalization. We ran these methods on the H3K27me3 data for ES and NP mouse cells provided by Mikkelsen *et al.* 2007 [3] (with whole cell extract (WCE) control library) and on the H3K27me3 data (of Broad Institute) for ES and GM12878 (replicate 1) from the human ENCODE project (ENCODE Project Consortium 2007 [4]). (GM12878 is a lymphoblastoid cell line produced from the blood of a female donor with northern and western European ancestry by EBV transformation [4].) Processing was done on individual chromosomes of the two libraries.

The methods other than ChIPnorm are as follows:

- *unit mean normalization*: is the standard Affymetrix scaling method for microarray data [1]. To normalize the bin values x_i of a library we calculated its trimmed mean $\bar{x}$ (the mean of the non-zero bins in the library) and then the normalized bin value is set to $x'_i = x_i/\bar{x}$. Finally a threshold (τ)

was used to classify bins as differential or not.

- *quantile normalization*: the two libraries are quantile normalized, and a fold change threshold (τ) is used to classify bins as differential or not.
- *MACS peak finder method*: Although MACS is a peak-finding software [5], we use it indirectly to find differential regions as follows: peaks for one library are detected by giving the other library as control, and the bins with peaks are considered as differential regions. The version of MACS software used is “macs14 1.4.1 20110622”.
- *ChIPDiff*: ChIPDiff method [2] is applied to find differential regions.
- *rank normalization*: the bin values of each of the libraries are sorted separately; the sorted lists are divided into 10 equal partitions, which we define as rank. Finally we compare the values of corresponding ranks at each bin value in both libraries. If the difference between the values is greater than a threshold ν then the bin is classified as differential.
- *RSEG method:* RSEG is a recently published method [6] to not only find peaks in histone modifications but to also identify differential regions (rseg-diff) between two histone modification ChIP-seq libraries.
- *two-stage unit mean normalization*: we removed the noisy bins using the first stage of the ChIPnorm method before applying the unit mean normalization and fold change classification.

For methods unit mean normalization, quantile normalization, rank normalization, two-stage unit mean normalization, ChIPnorm, the fold change ratios where calculated by adding +1 on the numerator and denominator before calculating the ratio so as to avoid the divide-by-zero case. For the sensitivity analysis using the ENCODE data the corresponding gene expression data (RPKM - Reads Per Kilobase of exon model per Million mapped reads) is obtained from the ENCODE Caltech RNA-seq database [4,7]. To calculate four-fold gene expression ratio for the ENCODE human data, the RPKM values of the two libraries were required to be normalized so that they are comparable to each other. So RPKM values of the library L_b was normalized by dividing it by the sum of all the RPKM values (of all genes) of library L_b and multiplying it by sum of all the RPKM values (of all genes) of library L_a. A small offset value of 5 was added to the RPKM values of each library before taking the fold change ratio so as to avoid division by zero or very small values. This is a common procedure and some other values of offset could also be chosen as it does not bias the results.

For the correlation with gene expression studies and the bivalent analysis studies, we classified the genes from Mikkelsen *et al.* 2007 [3] into the five groups (A-E) based on their increasing log-ratio of their expression levels in ES and NP cells. To ensure that each group has a good representation in terms of the number of genes, we created a histogram of the differential gene expression and divide them into $< -6\sigma$, -6σ to -2σ, -2σ to 2σ, 2σ to 6σ, $> 6\sigma$, from the mean, where σ is the standard deviation.

Results

Comparative Analysis

Table S1 shows the values of the five different thresholds T1, T2, T3, T4, T5, used in the sensitivity analysis (Fig. 7 of main paper).

Table S1. *Values of the various thresholds (T1, T2, T3, T4, T5) used for the various methods used in Fig. 7 of main paper.*

thresholds	unit-mean	quantile	MACS	ChIPDiff	RSEG	two-stage unit-mean	ChIPnorm
	τ	τ	p-val	τ	cdf	τ	τ
T1	3	11	10^{-2}	1.1	0.25	0.98	2
T2	5	13	10^{-4}	2	0.5	1.48	2.5
T3	7	15	10^{-6}	3	0.75	1.98	3
T4	9	17	10^{-8}	4	0.9	2.48	3.5
T5	11	19	10^{-10}	5	0.95	2.98	4

Differentially enriched regions along protein coding genes

Fig. F2 shows the relative enrichment of the regions identified by the ChIPnorm for human ES and GM12878 H3K27me3 ENCODE data along the various genomic features of protein coding genes (GENCODE v3c genes [8]). The regions, which are differentially enriched in ES cells (L1_enrich), are present along the 5' flanking end and initial exons of genes. This shows that these regions are mostly present in the promoter regions of genes. We also see that the regions that are differentially enriched in GM12878 cells (L2_enrich) are also present in the promoter regions of genes. However, regions, which are not differentially enriched. (non_differential) are absent along the promoter regions of genes. Therefore we provide evidence that the promoter regions of protein coding genes mainly contain differentially enriched regions and very few non-differential regions.

References

1. Bolstad BM, Irizarry RA, Astrand M, Speed TP (2003) A comparison of normalization methods for high density oligonucleotide array data based on variance and bias. Bioinformatics 19(2):185.

2. Xu H, Wei CL, Lin F, Sung WK (2008) An HMM approach to genome-wide identification of differential histone modification sites from ChIP-seq data. Bioinformatics 24(20):2344–2349.

3. Mikkelsen TS, Ku M, Jaffe DB, Issac B, Lieberman E, et al. (2007) Genome-wide maps of chromatin state in pluripotent and lineage-committed cells. Nature 448(7153):553–560.

4. ENCODE Project Consortium, Birney E, Stamatoyannopoulos JA, Dutta A, Guigo R, et al. (2007) Identification and analysis of functional elements in 1% of the human genome by the ENCODE pilot project. Nature 447(7146):799–816.

5. Zhang Y, Liu T, Meyer CA, Eeckhoute J, Johnson DS, et al. (2008) Model-based Analysis of ChIP-Seq (MACS). Genome Biol 9(9): R137.

6. Song Q, Smith AD (2011) Identifying dispersed epigenomic domains from ChIP-Seq data. Bioinformatics 27(6):870-1

7. Rosenbloom KR, Dreszer TR, Pheasant M, Barber GP, Meyer LR, et al. (2010) ENCODE whole-genome data in the UCSC Genome Browser. Nucleic Acids Res. 38(Suppl 1):D620-D625.

8. Harrow J, Denoeud F, Frankish A, Reymond A, Chen CK, et al. (2006) GENCODE: producing a reference annotation for ENCODE. Genome Biol. Suppl1:S4.1-9.

9. Buske OJ, Hoffman MM, Ponts N, Le Roch KG, Noble WS (2011) Exploratory analysis of genomic segmentations with Segtools. BMC Bioinformatics 12:415.

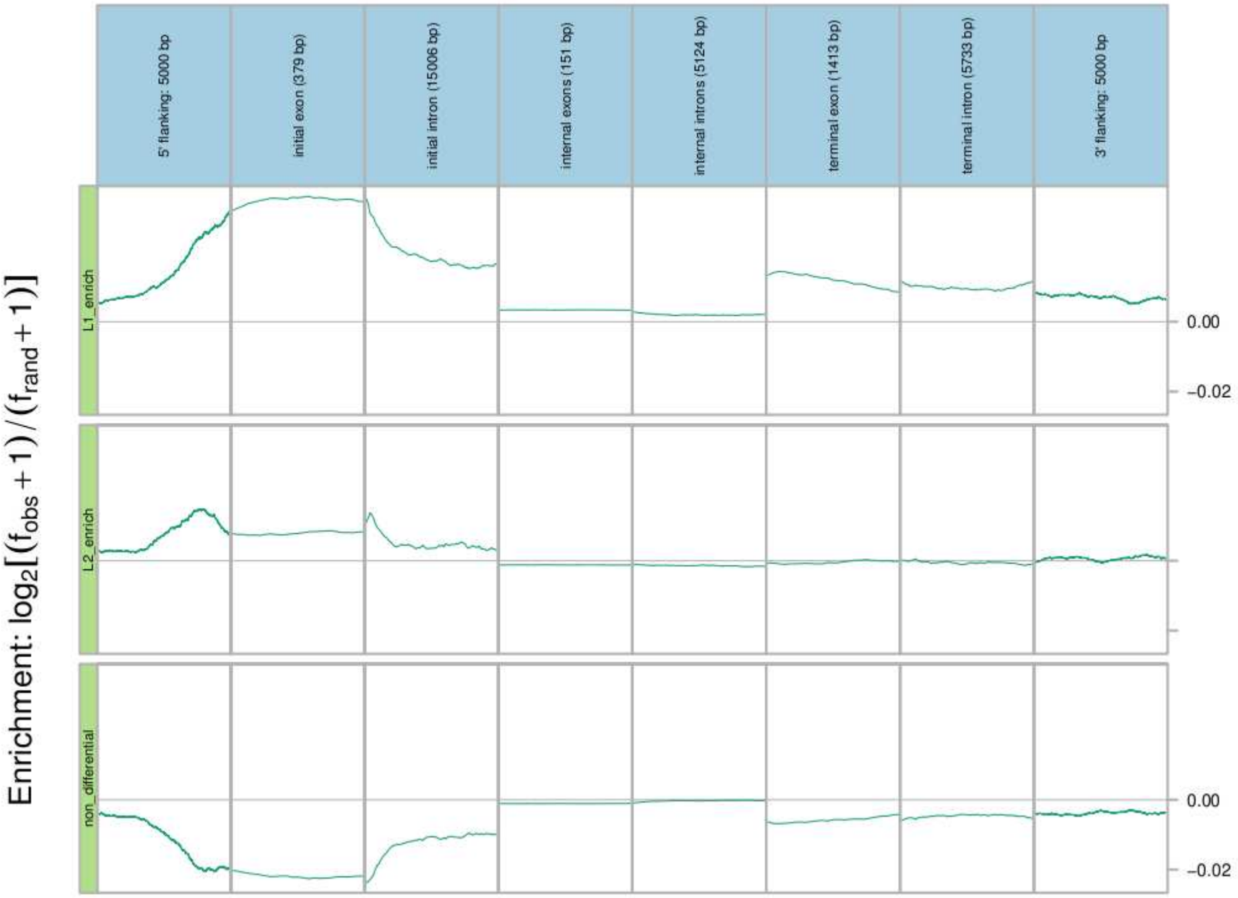

Figure F2. Feature aggregate plot of the differential/non-differential regions identified by the ChIPnorm method. Each row corresponds to a region from ChIPnorm and each column corresponds to a genomic feature of protein coding GENCODE genes. Curve inside a cell represents the relative frequency of overlap between the ChIPnorm identified regions and the genomic feature of GENCODE genes, when compared to a similar relative frequency of overlap that would occur by random chance. Figure created using Segtools [9].
